# Distinct forms of synaptic inhibition and neuromodulation regulate calretinin-positive neuron excitability in the spinal cord dorsal horn

**DOI:** 10.1016/j.neuroscience.2016.03.058

**Published:** 2016-06-21

**Authors:** K.M. Smith, K.A. Boyle, M. Mustapa, P. Jobling, R.J. Callister, D.I. Hughes, B.A. Graham

**Affiliations:** a School of Biomedical Sciences & Pharmacy, Faculty of Health, University of Newcastle, Callaghan, NSW, Australia; b Institute of Neuroscience Psychology, College of Medical, Veterinary & Life Sciences, University of Glasgow, Glasgow, UK

**Keywords:** ACSF, artificial cerebrospinal fluid, DH, dorsal horn, HC, holding current, mIPSCs, miniature inhibitory postsynaptic currents, PBS, phosphate-buffered saline, RMS, root mean square, sIPSCs, spontaneous inhibitory postsynaptic currents, glycine, GABA, noradrenaline, serotonin, enkephalin, pain

## Abstract

The dorsal horn (DH) of the spinal cord contains a heterogenous population of neurons that process incoming sensory signals before information ascends to the brain. We have recently characterized calretinin-expressing (CR+) neurons in the DH and shown that they can be divided into excitatory and inhibitory subpopulations. The excitatory population receives high-frequency excitatory synaptic input and expresses delayed firing action potential discharge, whereas the inhibitory population receives weak excitatory drive and exhibits tonic or initial bursting discharge. Here, we characterize inhibitory synaptic input and neuromodulation in the two CR+ populations, in order to determine how each is regulated. We show that excitatory CR+ neurons receive mixed inhibition from GABAergic and glycinergic sources, whereas inhibitory CR+ neurons receive inhibition, which is dominated by glycine. Noradrenaline and serotonin produced robust outward currents in excitatory CR+ neurons, predicting an inhibitory action on these neurons, but neither neuromodulator produced a response in CR+ inhibitory neurons. In contrast, enkephalin (along with selective mu and delta opioid receptor agonists) produced outward currents in inhibitory CR+ neurons, consistent with an inhibitory action but did not affect the excitatory CR+ population. Our findings show that the pharmacology of inhibitory inputs and neuromodulator actions on CR+ cells, along with their excitatory inputs can define these two subpopulations further, and this could be exploited to modulate discrete aspects of sensory processing selectively in the DH.

## Highlights

• CR+ spinal dorsal horn neurons form excitatory (Typical) and inhibitory (Atypical) subpopulations.
• Typical neurons received mixed (GABAergic and glycinergic) inhibition.
• Atypical neurons received inhibition dominated by glycine.
• Noradrenaline and serotonin evoke responses in Typical but not Atypical neurons.
• Enkephalins evoke responses in Atypical but not typical neurons.

## Graphical abstract

**Figure:**
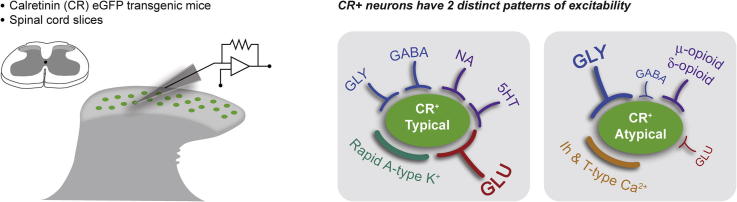

## Introduction

The dorsal horn (DH) of the spinal cord is a key region for processing sensory signals from skin, joints, muscle and viscera (Todd, 2010). A significant barrier to understanding how this processing takes place has been the substantial heterogeneity among the interneurons that populate the DH and the lack of information about how specific interneuron subpopulations participate in sensory processing (Graham et al., 2007). Fortunately, a number of technological advances are now allowing us to move from an ‘averaged view’ of DH interneuron function, to one that accounts for interneuron heterogeneity and defines the roles of specific neuron subpopulations and their connections. For example, several groups have studied GFP-labeled neurons as distinguished by their neurochemical phenotype (Hantman, 2004, Heinke et al., 2004, Zeilhofer et al., 2004, Hughes et al., 2012, Punnakkal et al., 2014, Smith et al., 2015). Other groups have used genetic ablation, or chemo-genetic activation/inactivation to show that certain interneuron subpopulations in the DH play precise roles in sensory processing under both normal and pathological conditions (Duan et al., 2014, Foster et al., 2015, Peirs et al., 2015, Petitjean et al., 2015). This effort is assembling information on how specific populations of DH interneurons interact in spinal circuits to shape sensory experience (Smith et al., 2014).

One DH interneuron population, which has been subjected to such analyses, expresses the calcium binding protein calretinin. These calretinin expressing (CR+) neurons are largely excitatory and have been implicated in a polysynaptic circuit that links innocuous tactile input with nociceptive circuitry (Peirs et al., 2015). This link relays input from VGLUT3+ interneurons to nociceptive circuits and is responsible for mechanical hypersensitivity/allodynia. These findings are also supported by work where CR+ neurons have been selectively ablated (Duan et al., 2014). Mice with ablated CR+ neurons showed enhanced hind paw withdrawal thresholds to noxious mechanical stimuli, but no change in motor coordination, light touch, thermal, pinprick, and pinch stimulation thresholds. Together, these studies suggest CR+ neurons can link innocuous tactile information with nociceptive circuits and cause allodynia. Under normal conditions this connection is ‘silent’ because light touch stimulation does not cause pain, however, certain pathologies can activate this pathway. Thus reducing or silencing the activity of CR+ interneurons could relieve allodynia, whereas modulating their properties would alter responses in chronic pain states.

Our group has recently assessed selected properties of CR+ neurons in the DH (Smith et al., 2015). Surprisingly, despite the broad use of calretinin as a *de facto* marker for excitatory interneurons, we identified excitatory and inhibitory populations of CR+ neurons. This was based on: (1) CR+ neurons being labeled with PAX2, a marker of inhibitory interneurons in the DH; and (2) these neurons possessing the morphological features typical of inhibitory DH interneurons. We used the terms Typical and Atypical to differentiate the two CR+ populations, based on their incidence. Typical CR+ neurons constitute ∼90% of the sample and represent the excitatory population whereas Atypical CR+ neurons comprise the remaining 10% and are inhibitory. Typical CR+ neurons were morphologically diverse and exhibit central, vertical and radial morphologies, whereas Atypical CR+ neurons have islet-cell like morphology. In addition, the intrinsic properties and excitatory synaptic input to the two CR+ populations exhibit opposing excitability profiles (Smith et al., 2015). Typical CR+ neurons receive very strong excitatory drive but also express I_A_ potassium currents and delayed AP discharge, which limits excitability. Conversely, Atypical CR+ neurons receive only weak excitatory drive but express both I_h_ and T-type calcium currents, and exhibit more ‘excitable’ tonic firing or initial bursting forms of AP discharge. Based on these findings we proposed two different CR+ neuron populations exist in the DH, and that these have distinct roles in normal and dysfunctional sensory processing. Here, we further test this hypothesis by examining inhibitory inputs to both Typical and Atypical CR+ neurons as well as their responsiveness to several neuromodulators that are known to be important in sensory processing. This information is important for a more complete understanding of how the two CR+ neuron subpopulations participate in spinal sensory processing.

## Experimental procedures

### Animals

All procedures were approved by the Animal Care and Ethics Committee at the University of Newcastle. Anatomical studies were performed on C57Bl/6J mice (body weights 22 and 24 g), whereas all electrophysiological studies were carried out on transgenic mice (both sexes, body weight 22–30 g) that expressed enhanced green fluorescent protein (eGFP) under control of the calretinin promoter (CReGFP: Caputi 2008). The CReGFP line was generated by Prof Hana Monyer and bred with her permission at the UoN on the C57Bl/6J background. Mice were housed 6/cage in temperature- and humidity-controlled conditions on a standard 12-h–12-h light–dark cycle with *ad libitum* access to food and water.

### Tissue preparation for anatomical studies

Two adult male mice were deeply anesthetized with pentobarbital (20 mg i.p.) and perfused through the left ventricle with 4% depolymerized formaldehyde in 0.1 M phosphate buffer. Following perfusion fixation, the L4 spinal segments were removed and cut into 60-μm-thick transverse sections with a Vibratome. These sections were incubated in cocktails of primary antibodies containing either rabbit anti-calretinin (1:1000 dilution; Swant, Belinoza, Switzerland) and mouse anti-NeuN to label all neurons (1:1000; Millipore, Watford, UK), or goat anti-calretinin (1:1000 dilution; Swant, Belinoza, Switzerland) and rabbit anti-Pax2 (1:1000; Invitrogen, Paisley, UK) for 48 h. Sections were then incubated in species-specific secondary antibodies conjugated to Alexa 488, Alexa 647 or Rhodamine for 24 h, and followed by counterstaining with DAPI. Between four sections from each animal were scanned with a x40 oil-immersion lens, 1-μm z-step on a Zeiss LSM710 confocal microscope with Argon multi-line, 405-nm diode, 561-nm solid state and 633-nm HeNe lasers.

All antibodies used in immunofluorescence protocols were diluted in phosphate-buffered saline (PBS) containing 0.3% Triton X-100. Each primary antibody has been characterized fully: rabbit anti-calretinin (Schwaller et al., 1994); goat anti-calretinin (Schiffmann et al., 1999); rabbit anti-Pax2 (Dressler and Douglass, 1992); mouse anti-NeuN (Mullen et al., 1992, Todd et al., 1998). All incubations were carried out at 4 °C.

### Stereological analysis and cell counts

The resulting scans from each section were analyzed using a modified dissector method as described previously (Sardella et al., 2011). Briefly, to determine the proportion of DH neurons that express CR-immunolabeling, NeuN and DAPI staining were initially viewed with Neurolucida for Confocal software (MicroBrightField, Colchester, VT, USA). In each z-series, the 5th optical section was designated as the reference section and the 25th as the look-up section. Every optical section in the series was then viewed and the locations of cell bodies of all neurons (identified by the presence of both NeuN and DAPI staining) that were present in the reference section, or appeared in subsequent sections, were plotted onto an outline of the gray matter. All of those cells with nuclei that were still present in the look-up section were then excluded, leaving only those for which the bottom surface of the nucleus was located between the reference and look-up sections. The channel corresponding to CR-immunostaining was then viewed, and the presence or absence of CR immunoreactivity in each of the selected cells was noted. To determine the proportion of CR-immunoreactive neurons that were inhibitory, CR-IR cells were first plotted as described above for identifying the cell bodies of neurons, before noting the presence or absence of Pax2 labeling in these cells. The outline of the DH and boundaries between laminae I, II and III were determined from corresponding images from the Allen Brain Atlas (http://mousespinal.brain-map.org/). In each case, these outlines were verified further by measuring a line 100 μm from the border between the DH gray matter and the white matter to demark lamina I, and by outlining the ventral aspect of the CR-IR plexus to delineate the border between laminae II and III.

### Slice preparation for electrophysiology experiments

Spinal cord slices were prepared from 21 CReGFP mice using previously described methods (Graham et al., 2003). Briefly, animals were anesthetized using ketamine (100 mg/kg i.p.) and decapitated. Using a ventral approach, the lumbosacral enlargement of the spinal cord was rapidly dissected and placed in ice-cold sucrose-substituted artificial cerebrospinal fluid (ACSF) containing (in mM): 250 sucrose, 25 NaHCO_3_, 10 glucose, 2.5 KCl, 1 NaH_2_PO_4_, 1 MgCl_2_ and 2.5 CaCl_2_. Parasagittal slices (from L3-L5 segments; 300-μm-thick) were obtained using a vibrating microtome (Leica VT-1000S, Heidelberg, Germany) and transferred to an interface incubation chamber containing oxygenated ACSF (118 mM NaCl substituted for sucrose). Slices were allowed to equilibrate for 1 h at room temperature (22–24 °C) prior to recording.

### Electrophysiology

Slices were transferred to a recording chamber and continually superfused (bath volume 0.4 mls; exchange rate 4–6 bath volumes/min) with ACSF bubbled with Carbanox (95% O_2_ and 5% CO_2_) to achieve a pH of 7.3–7.4. Recordings were obtained at room temperature (21–24 °C) and neurons were visualized using near-infrared differential interference contrast optics. CReGFP-positive neurons were identified under fluorescence using a FITC filter set (488-nm excitation, 508-nm emission filters). Recordings were obtained from neurons located within or dorsal to the substantia gelatinosa. This area is identified by its translucent appearance in spinal cord slices and contains a clearly discernible plexus of CReGFP-positive neurons. Our parasagittal slicing approach allowed selective targeting of the *Typical* and *Atypical* CR+ populations as putative *Atypical CR+* neurons could be recognized by their extensive dendritic projections in the rostrocaudal plane. In contrast, the dendritic arbors of *Typical* CR+ neurons were limited to a more restricted area around the neuron’s soma. Inhibitory synaptic inputs were recorded in the presence of the AMPA/kainate receptor antagonist 6-cyano-7-nitroquinoxaline-2,3-dione (CNQX – 10 μM), to abolish excitatory inputs, from a holding potential of −70 mV. Patch pipettes (4–8 MΩ) were filled with a cesium chloride-based internal solution containing (in mM): 130 CsCl, 10 Hepes, 10 EGTA, 1 MgCl2, 2 ATP and 0.3 GTP (pH adjusted to 7.35 with 1 M CsOH). A potassium gluconate-based internal solution containing (in mM): 135 C_6_H_11_KO_7_, 6 NaCl, 2 MgCl_2_, 10 HEPES, 0.1 EGTA, 2 MgATP, 0.3 NaGTP, pH 7.3 (with KOH) was used in experiments that examined responses to neuromodulators (holding potential of −70 mV). Neurobiotin (0.2%) was included in both internal solutions for post-hoc confirmation of neuronal morphology (Vector Laboratories, Peterborough, UK).

### Neurobiotin labeling and recovery for morphological analysis

For analysis of neuron morphology slices were immersion fixed at the end of a recording session for at least 24 h in 4% depolymerized formaldehyde or 4% depolymerized formaldehyde with 0.2% glutaraldehyde. Slices were washed in PBS, incubated for 24 h in Avidin-rhodamine (diluted 1:1000; Jackson ImmunoResearch, West Grove, PA, USA), and mounted on glass slides. The morphology of filled neurons was reconstructed using confocal image stacks collected using a 20× lens and 2-μm z-separation. Where recovered neurons exceeded the visual field captured at 20×, overlapping fields were scanned and merged. Image stacks were then stitched together using Zen 2010 software (Carl Zeiss MicroImaging) and viewed in Adobe Photoshop 11.0 (Adobe Systems, San Jose, CA, USA). For each neuron, all labeled profiles were selected and pasted onto a black background as described previously (Yasaka et al., 2010).

### Drugs

Tetrodotoxin (TTX) was purchased from Alomone Labs (Jerusalem, Israel). All other drugs were purchased from SIGMA. Drugs were prepared and stored at 1000× final concentration and diluted in bath perfusate prior to application.

### Data analysis

Only recordings from neurons with a series resistance <30 MΩ (filtered at 5 kHz) that remained stable over the recording duration (<20% change) were retained for offline analysis using Axograph software. In recordings of inhibitory synaptic input (spontaneous inhibitory postsynaptic currents (sIPSCs) and miniature inhibitory postsynaptic currents (mIPSCs)) were detected and captured using a sliding template method (a semi-automated procedure within Axograph software package) (Clements and Bekkers, 1997). Captured events were inspected individually and excluded from the analysis if they contained over-lapping currents or had an unstable baseline before the rise or after the decay phase of the current. Data were rejected if a significant trend was evident in either amplitude or instantaneous frequency of detected events over the time course of the experiment. Analyses were performed on averaged sIPSCs and mIPSCs, generated by aligning the rising phase of all accepted events. Peak amplitude, rise-time (calculated over 10–90% of peak amplitude) and decay time constant (calculated over 20–80% of the decay phase) were obtained using automated procedures within the Axograph analysis program. Average sIPSC and mIPSC frequency were obtained by dividing the number of captured events by the analysis duration in seconds. In addition to inhibitory synaptic currents, we also tested for the presence of tonic GABA_A_ and glycine currents by comparing the holding current (HC) and root mean square (RMS) noise in mIPSC recordings prior to and following bath application of bicuculline (10 μM) or strychnine (1 μM) in *Typical* and *Atypical* CR+ neurons.

In recordings that assessed neuromodulator responses the mean baseline HC was measured prior to neuromodulator application and then at the peak response, with the difference reported as response amplitude. In addition, a test pulse delivered throughout the recording was used to calculate neuronal input resistance before, during, and after neuromodulator application. *Typical CR+* neurons were easily identified in neuromodulator recordings by their high frequency of sEPSCs and the presence of A-type potassium currents during depolarizing step injections from hyperpolarized membrane potentials. In contrast, *Atypical CR+* neurons had low-frequency sEPSCs and they did not express the A-type potassium current. These criteria could not be assessed during sIPSC recordings and therefore the initial assignment as *Typical* or *Atypical CR+* was based on the neuron morphology in the acute slices, where *Atypical CR+* neurons have extensive and clearly defined islet cell-like morphology. This initial categorization of CR+ neurons was verified in a subset of Neurobiotin-filled neurons (*n* = 20) after recording sessions. *Typical* and *Atypical CR+* neurons were differentiated by examining their rostrocaudal dendritic arborizations, then subsequently quantifying the ratio of their rostrocaudal to dorsoventral extensions.

### Statistics

Statistical analysis was carried out using SPSS v10 (SPSS Inc. Chicago, IL). Student *t*-tests were used to compare variables across Typical and Atypical CR+ recordings. Data that failed Levene’s test for homogeneity of variance were compared using the non-parametric Kruskal–Wallis test. Statistical significance was set at *p* < 0.05. All values are presented as mean ± SEM.

## Results

### Distribution of calretinin-expressing cells

**Fig. 1:**
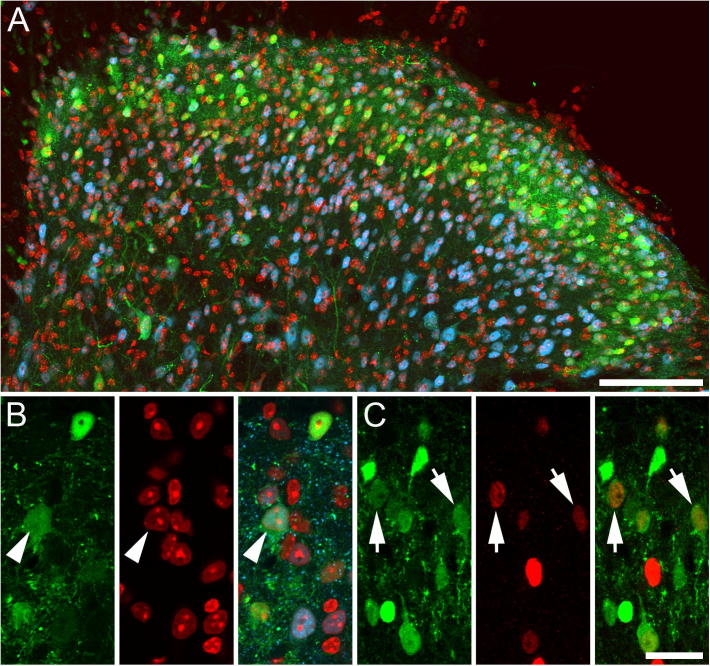
Distribution of CR-expressing neurons in the mouse spinal dorsal horn. (A, B) CR-IR neurons (green) are most common in laminae I and II of the mouse spinal dorsal horn, and account for approximately 30% of all neurons in these laminae. The nuclear stain DAPI (red) and immunolabeling for the selective neuronal marker NeuN (blue) were used to plot the position of all neurons for use in the stereological analysis to determine the proportion of neurons in laminae I and II that express CR (green). CR-IR neurons are marked with an arrowhead. (C) The proportion of CR cells (green) in laminae I and II that express Pax2 (red), a marker of inhibitory interneurons in the spinal dorsal horn, was also determined using the dissector method. Inhibitory CR-IR cells are marked with an arrow. Fig.1A is an image projected from 14 optical sections at 1 μm z-separation, Fig.1B, C are projections of three optical sections at 1 μm z-separation. Scale bars: A=100 μm; B and C=20 μm. (For interpretation of the references to colour in this figure legend, the reader is referred to the web version of this article.)

The distribution of CR-immunoreactive (CR-IR) cells was as described previously (Smith et al., 2015), with immunolabeled cells being most common in lamina II (Fig. 1). Stereological analysis showed that CR-IR cells accounted for ∼30% of all neurons in laminae I and II (622 of 2057 neurons, and 327 of 1021 neurons, respectively), and that Pax2 was expressed in 15% of CR-immunolabeled cells in these laminae (54 of 403 CR-IR cells, and 90 of 514 CR-IR cells, respectively).

### Morphology of CR-positive populations

**Fig. 2:**
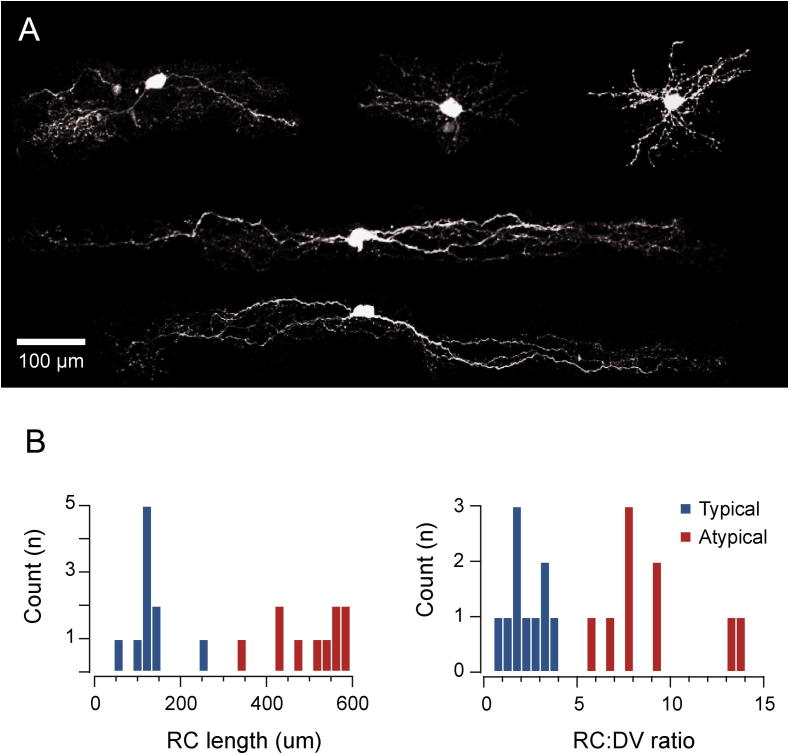
*Typical and Atypical CR-positive neurons exhibit distinct morphologies.* (A) Images show representative examples of Neurobiotin-filled CR+ neurons in sagittal section. Note the relatively compact morphology of Typical CR+ neurons (upper three images) versus the distinct expansive islet cell-like morphology of Atypical CR+ neurons (lower two images). (B) Histograms showing the distribution of rostrocaudal (RC) dendritic length and the ratio of rostrocaudal/dorsoventral length for *Typical* and *Atypical* CR+ neurons putatively identified in acute slices. Note the of *Typical CR+* neuron values for RC length and RC:DV ratio are clustered to the left of the distribution, whereas *Atypical CR+* values are all found on the right. This separation confirms that CR+ neuron subpopulations can be clearly identified in acute spinal cord slices by examining neuron morphology under GFP fluorescence.

The cesium-based internal solution we used for studying inhibitory synaptic transmission did not allow the use of functional properties including AP discharge and EPSC frequency to differentiate *Typical* and *Atypical* CR+ neurons. Therefore, comparisons of inhibitory synaptic input were carried out on *Typical* and *Atypical* CR+ neurons identified according to their dendritic morphology in acute parasagittal slices, validated in a subset of successfully recovered neurons. Fig. 2A shows examples of Neurobiotin-recovered CR+ neurons, all of which were found in lamina II. The *Typical* CR+ neurons (*n* = 10) exhibit restricted radial, vertical and central morphologies. In contrast, *Atypical* CR+ neurons (*n* = 10) exhibit distinct expansive islet cell-like morphology. This was validated by group comparisons of maximum rostrocaudal length (127.5 ± 15.5 μm vs. 502.8 ± 26.3 μm, *p* < 0.05) and rostrocaudal to dorsoventral ratio (2.2 ± 0.3 vs. 9.6 ± 0.9, *p* < 0.05) in filled neurons. The data show a clear bimodal distribution and confirm that *Atypical* and *Typical* CR+ neurons can be distinguished based on their morphology in acute slices (Fig. 2B).

### Inhibitory input to CR-positive populations

**Fig. 3:**
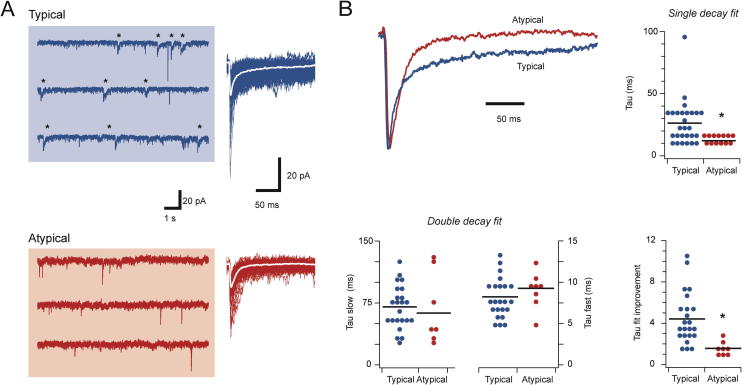
*Synaptic inhibition differs in Typical and Atypical CR-positive neurons.* (A) Left traces show continuous sIPSC recordings from *Typical* and *Atypical CR+* neurons. Asterisks highlight sIPSCs with slow decay times – a feature of *Typical* but not *Atypical CR+* neurons. Right, overlaid and aligned sIPSCs showing their amplitudes and time courses. Note the longer time courses in the sIPSCs from the *Typical CR+* neuron. (B) Averaged sIPSCs from A, normalized to the same amplitude, comparing sIPSC time course between *Typical* and *Atypical CR+* neurons. Top right plot compares group data for sIPSC decays fitted with a single exponential. sIPSCs were markedly faster in *Atypical CR+* neurons. Bottom left plots compare fast and slow decay time constants for sIPSCs when fit with a double exponential. The slow and fast components were similar in both neuron types. Bottom right plot compares the improvement in sIPSC decay fit between single and double exponentials, quantified as the ratio of the sum of squares error for double and single exponential fits. Values of 1 indicate no improvement, whereas values greater than 1 indicate fit is better for double exponential.

sIPSCs were recorded from *Typical* and *Atypical* CR+ populations (*n* = 25 and 14, respectively, Fig.3A). The frequency of sIPSCs was similar (0.67 ± 0.14 vs. 0.31 ± 0.09 Hz, *p* = 0.08), as was sIPSC amplitude (34.3 ± 2.3 vs. 25.8 ± 3.1 pA, *p* < 0.05) and rise time (1.93 ± 0.16 vs. 2.00 ± 0.19 ms, *p* = 0.79). In contrast, when the decay phases of averaged sIPSCs were fit with a single exponential, the resulting decay time constant was markedly slower in *Typical* versus *Atypical* CR+ recordings (25.74 ± 3.83 vs. 12.14 ± 0.81 ms, respectively, *p* < 0.05). As GABA, glycine, or both can mediate fast synaptic inhibition in the spinal cord, and because these two transmitter systems exhibit characteristically different decay times, we also attempted to fit the decay phase of averaged sIPSCs with double exponentials. This exercise showed *both* fast and slow exponentials could be fit to 90% of the sIPSCs for *Typical* CR+ recordings (23/25), but only in 60% of *Atypical* CR+ recordings (8/14). Furthermore in neurons with a double exponential fit the goodness of fit versus a single exponential, assessed as the ratio of the ‘sum of squares error’ for single versus double fits, was significantly greater in *Typical* CR+ neurons (4.39 ± 0.53 vs 1.54 ± 0.18, respectively, *p* < 0.01). This further supports our claim that biphasic sIPSC decays were more prominent in *Typical* CR+ neurons. Despite these differences, the slow (70.10 ± 5.52 vs. 62.70 ± 15.84 ms, *p* = 0.58) and fast (8.19 ± 0.53 vs. 9.22 ± 0.73 ms, *p* = 0.31) decay time constant components were similar in *Typical* and *Atypical* CR+ neurons. This suggests the same underlying processes (ie, presumably GABAergic and glycinergic transmission) contribute to the sIPSCs in each neuron type, though to differing extents. It also suggests *Typical* CR+ neurons receive mixed inhibition with a greater contribution from a slower decaying, GABAergic component, whereas inhibitory drive to *Atypical* CR+ neurons is dominated by a faster decaying glycinergic component.

**Fig. 4:**
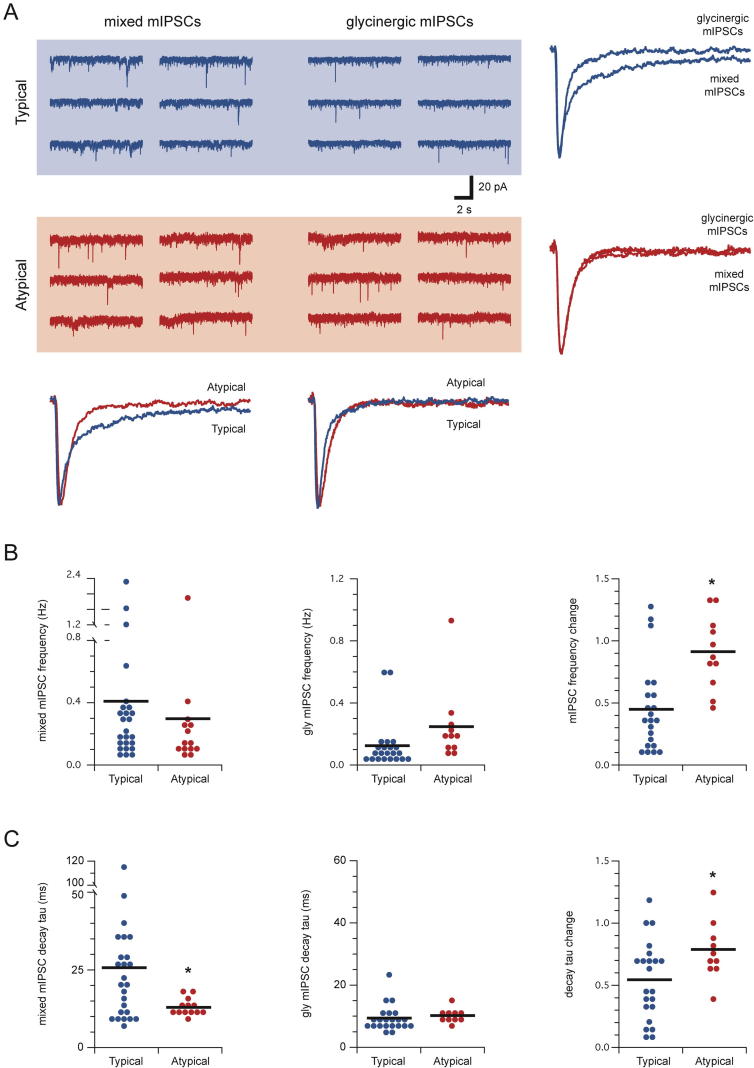
*Typical CR-positive neurons receive a combination of ‘mixed’ GABAergic and glycinergic inhibition whereas glycinergic inhibition dominates in Atypical CR+ neurons*. (A) Traces show continuous recordings of miniature inhibitory postsynaptic currents (mIPSCs) from Typical (upper) and Atypical CR+ (lower) neurons; before (mixed mIPSCs, left) and after (glycinergic mIPSCs, right) blocking GABA_A_ergic mIPSCs with bicuculline (conc 10 μM). Overlaid currents (right and below) compare averaged mixed mIPSCs and glycinergic mIPSCs from *Typical* and *Atypical CR+* neurons. Note mixed mIPSCs decay is significantly slower than glycinergic mIPSCs in Typical CR+ recordings, whereas Atypical CR+ mixed and glycinergic mIPSC decays are similar. (B) Plots comparing group data for mixed mIPSCs frequency, glycinergic mIPSCs frequency, and change in frequency for *Typical* and *Atypical CR+* neurons in the absence and presence of bicuculline. Although the frequency of mixed and glycinergic mIPSCs was similar in the two populations, the change in mIPSC frequency differed significantly – mIPSC frequency was reduced by ∼50% in *Typical CR+* neurons after the addition of bicuculline, whereas mIPSC frequency was similar under both conditions in *Atypical CR+* neurons. (C) Left and middle plots compare single decay time constants fitted to mixed mIPSCs and glycinergic mIPSCs. Right plots show the change in mIPSC decay under the two recording conditions (mixed vs glycinergic mIPSCs) in *Typical* and *Atypical CR+* neurons. The decay of mixed mIPSCs was slower in *Typical CR+* neurons, whereas glycinergic mIPSC decay was similar in the two populations. mIPSC decay times in *Typical CR+* neurons, when expressed as a change pre versus post bicuculline, were more dramatically affected than those from Atypical CR+ neurons (∼50% vs 20% change).

To further assess the relative contribution of GABAergic and glycinergic inhibition to CR+ neurons we also recorded mIPSCs from the sample (*n* = 25 and 14, Typical and Atypical neurons respectively) and then tested the sensitivity of these currents to the GABA_A_-receptor antagonist bicuculline (10 μM). Consistent with the sIPSC data, mIPSC frequency (0.41 ± 0.11 vs. 0.29 ± 0.13 Hz, respectively, *p* = 0.51), amplitude (26.8 ± 1.6 vs. 23.2 ± 2.9 pA, respectively, *p* = 0.25), and rise times (1.98 ± 0.14 vs 1.81 ± 0.08 ms, respectively, *p* = 0.40) were similar in *Typical* and *Atypical* CR+ neurons prior to bicuculline exposure (Fig.4A). However, single exponentials fitted to mIPSC decay phases showed significantly slower time constants in *Typical* versus *Atypical* CR+ recordings (25.38 ± 4.43 vs. 12.77 ± 0.73 ms, respectively, *p* < 0.05), whereas double exponential fits yielded similar slow (77.71 ± 10.42 vs. 104.28 ± 68.88 ms, respectively, *p* = 0.58) and fast (9.61 ± 1.42 vs. 7.50 ± 0.64 ms, *p* = 0.24) decay time constants in both populations. Like our analysis of sIPSCs it appears a more substantial GABAergic component exists in the inhibition onto *Typical* compared to *Atypical* CR+ neurons. Consistent with this interpretation, mIPSC properties were affected differently by abolishing the GABAergic mIPSCs with bicuculline (Fig.4B). As expected, mIPSC frequency was reduced by >50% following bicuculline exposure in *Typical* CR+ neurons (0.43 ± 0.12 vs. 0.12 ± 0.03, respectively, *p* < 0.01), whereas mIPSC frequency in *Atypical* CR+ neurons was not altered (0.35 ± 0.16 vs. 0.26 ± 0.07 Hz, respectively, *p* = 0.32). Similarly, the decay phase of mIPSCs decreased by >50% in *Typical* CR+ recordings after the addition of bicuculline (26.12 ± 4.96 vs. 9.34 ± 0.94 ms, respectively, *p* < 0.01), whereas mIPSC decay time constant in *Atypical* CR+ neurons remained similar in bicuculline (13.42 ± 0.85 vs. 10.16 ± 0.74 ms, respectively, *p* = 0.11).

In contrast to our analysis of synaptic inhibition, we saw no evidence for tonic inhibition in either *Typical* or *Atypical* CR+ neurons. The level of HC and RMS noise did not change after the addition of bicuculline in either *Typical* (HC: −56.6 ± 5.1 vs. −56.4 ± 4.8 pA, *p* = 0.90; RMS: 86 ± 12 vs 83 ± 11 pA, *p* = 0.89; *n* = 11) or *Atypical* (HC: −42.2 ± 3.2 vs. −44 ± 2.8 pA, *p* = 0.43; RMS: 64 ± 113 vs. 61 ± 12 pA, *p* = 0.42; *n* = 6) neurons, indicating neither population expresses tonic GABA_A_ergic currents. Likewise, HC and RMS noise did not change after the addition of strychnine in either *Typical* (HC: −66.6 ± 8.6 vs. −65.3 ± 8.6, *p* = 0.87; RMS: 53 ± 13 vs. 44 ± 17 pA, *p* = 0.23; *n* = 3) or *Atypical* (HC: −46.2 ± 8.5 vs. −48.9 ± 9.4 pA, *p* = 0.26; RMS: 125 ± 33 vs. 109 ± 37 pA, *p* = 0.14; *n* = 4), indicating neither population expresses tonic glycinergic currents. Together, these analyses confirm GABAergic synaptic inhibition plays a more significant role in the *Typical* population, glycinergic synaptic inhibition dominates in the *Atypical* CR+ neurons, and neither population exhibits tonic inhibitory currents.

### Neuromodulator responses in CR-positive populations

**Fig. 5:**
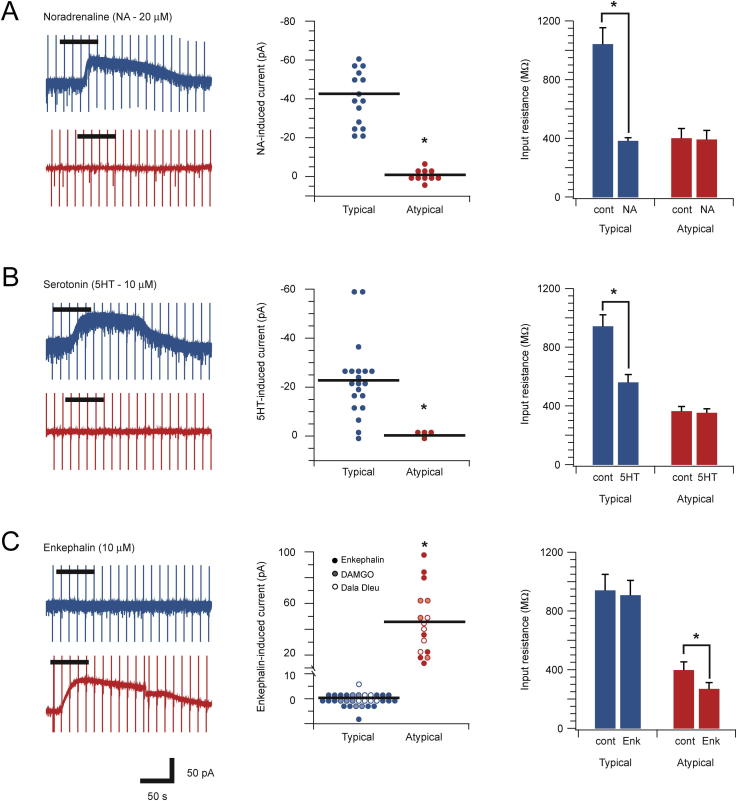
*Typical and Atypical CR-positive neurons respond differently to neuromodulators*. (A–C) Left traces show representative recordings from Typical and Atypical CR+ neurons during bath application (black bar above traces) of noradrenaline (NA – 20 μM), serotonin (5HT – 10 μM), and enkephalin (10 μM), respectively. Middle plots compare group data for peak membrane current amplitude during neuromodulator exposure in both neuron types. Right plots compare neuromodulator effects on neuronal input resistance. (A) Noradrenaline exposure evoked outward currents and reduced input resistance in all *Typical CR+* neurons but had no effect in *Atypical CR+* neurons. (B) Serotonin exposure evoked outward currents and reduced input resistance in most *Typical CR+* neurons but had no effect on *Atypical CR+* neurons. (C) Enkephalin exposure evoked outward currents and reduced input resistance in all *Atypical CR+* neurons, but not in *Typical CR+* neurons (filled symbols). The opioid receptor agonists DAMGO (shaded symbols) and DADLE (open symbols) also evoked outward currents in *Atypical* but not in *Typical CR+* neurons. These experiments indicate *Atypical CR+* neurons express mu- and delta-opioid receptors.

Various neuromodulators including noradrenalin, serotonin, and enkephalin have well-documented effects on DH neurons. To address how these neuromodulators influence CR+ neurons we examined their effects on the *Typical* and *Atypical* CR+ populations (Fig. 5). Bath application of noradrenalin (20 μM) induced an outward current (42.9 ± 4.3 pA) in all *Typical* CR+ neurons tested (15/15). None of the *Atypical* CR+ neurons exhibited a noradrenalin-induced current (0/10). Furthermore, the input resistance of *Typical* CR+ neurons was significantly reduced during noradrenalin exposure (1042 ± 113 vs. 382 ± 22 MΩ, *p* < 0.001), whereas input resistance in *Atypical* CR+ neurons was unchanged (401 ± 67 vs. 392 ± 62 MΩ, *p* = 0.41). A similar response profile was induced by bath-applied serotonin (10 μM). The majority (18/20) of *Typical* CR+ neurons exhibited outward currents during bath serotonin (22.8 ± 3.5 pA), which were not observed in *Atypical* CR+ neurons (0/4). Input resistance also fell in *Typical* CR+ neurons during serotonin exposure (942 ± 79 vs. 560 ± 54 MΩ, *p* < 0.001), but was unchanged in *Atypical* CR+ recordings (363 ± 33 vs. 352 ± 29 MΩ, *p* = 0.063). Bath application of enkephalin (10 μM) did not evoke whole-cell currents in *Typical* CR+ neurons (0/18) nor did it change input resistance (940 ± 110 vs. 907 ± 102 MΩ, *p* = 0.17). In contrast, enkephalin induced robust outward currents (54.4 ± 15.6 pA) in *Atypical* neurons (7/7) and significantly reduced input resistance (397 ± 57 vs. 268 ± 44 MΩ, *p* < 0.05). We also applied other opioid agonists to *Typical* (*n* = 7) and *Atypical* (*n* = 5) *CR+* neurons. The mu-opioid receptor agonist [D-Ala2, N-MePhe4, Gly-ol]-enkephalin (DAMGO) and the delta-opioid receptor agonist [D-Ala2, D-Leu5]-Enkephalin (DADLE) had no effect on *Typical CR+* neurons, but evoked outward currents (43.4 ± 9.5 and 39.1 ± 4.6 pA, respectively) in all *Atypical CR+* neurons. Together, these data suggest that *Atypical CR+* neurons express both mu- and delta-opioid receptors.

## Discussion

This study builds on previous work where we described two functionally distinct populations of CR+ interneurons in the mouse superficial DH (Smith et al., 2015). The *Typical* CR+ population exhibits morphological and electrophysiological properties consistent with glutamatergic, excitatory interneurons, while the *Atypical* CR*+* population has properties widely associated with inhibitory DH neurons. *Typical* CR+ neurons receive strong ongoing excitatory synaptic input but have low levels of intrinsic excitability (i.e. AP discharge). Conversely, *Atypical* CR+ neurons receive only weak ongoing excitatory input, but have high intrinsic excitability. Based on these findings we proposed *Typical* and *Atypical* CR+ neurons would process sensory information in very different ways. We have now undertaken a stereological analysis to determine the relative proportion of each population and show that CR+ interneurons constitute ∼30% of neurons in the SDH (lamina I and II). Furthermore, ∼15% of these are inhibitory and therefore this unbiased approach suggests that Typical (excitatory) CR+ neurons make up ∼25% of all SDH neurons and the Atypical (inhibitory) CR+ neurons constitute ∼4% of the population. We also explored the regulation of these two types of CR+ interneurons by examining their inhibitory synaptic input and response to neuromodulators known to be important in spinal sensory processing. Our results show that the *Typical* CR+ population receives mixed glycinergic and GABAergic inhibition, whereas *Atypical* CR+ neurons receive inhibition dominated by glycine. The *Typical* CR+ population responds to noradrenaline and serotonin but not enkephalin, whereas *Atypical* CR+ neurons respond to encephalin, but not noradrenaline or serotonin. These findings support our hypothesis that *Typical* and *Atypical* CR+ neurons form two distinct populations and provide additional insights into how the activity of these two CR+ interneuron types are regulated during sensory processing.

The importance of synaptic inhibition for spinal sensory processing has been a major focus in pain research since publication of the Gate Control Theory of Pain (Melzack and Wall, 1965). Numerous studies have subsequently demonstrated that inhibitory dysfunction, usually dis-inhibition, is crucial in a range of pathological pain states (Zeilhofer et al., 2012). Importantly, both GABA and glycine mediate fast inhibitory synaptic inhibition in the DH, and experimental manipulation of each transmitter can alter sensory processing and either generate or alleviate pain-related behaviors in rodent pain models (Ishikawa et al., 2000, Knabl et al., 2008). At the cellular level, key functional differences between GABAergic and glycinergic synapses lie in the time course and pharmacology of the currents they mediate (Anderson et al., 2009). Specifically, glycinergic synaptic currents exhibit fast decay times and simple pharmacology, whereas GABAergic current decay time courses are slower but are modulated by large number of endogenous and exogenous compounds (Callister, 2010). Thus, glycinergic synapses are thought to be best suited for regulation of rapid, time dependent and coordinated inhibition. Conversely, GABAergic synapses are more likely to be involved in time dependent summation that provides sustained inhibition.

The marked differences between GABAergic and glycinergic currents and the role they play in synaptic function are strongly influenced by receptor subunit composition. For example, GABAergic receptor composition varies considerably within the DH, with strong expression of α2, α3, α5, β2, β3, and γ2 subunits in superficial laminae (Bohlhalter et al., 1996, Paul et al., 2012). The functional importance of this subunit diversity is highlighted by work, which shows the α2 subunit is critical for the potentiating effect of benzodiazepines at GABAergic synapses. Receptors containing the α2 subunit also have faster kinetics (i.e. short decay times) than those containing α3 subunits. The faster α2 subunit containing GABA receptors are thought to be more prevalent on excitatory DH interneurons whereas α3 subunit containing receptors are more prevalent on inhibitory interneurons. Likewise, subunit composition has also proven important for glycine receptor function in the DH. Most notably, α3 subunit containing receptors are preferentially expressed in superficial laminae (I-II) whereas α1 subunit containing receptors are expressed more uniformly throughout the spinal cord (Anderson et al., 2009, Graham et al., 2011). Glycine receptors containing the α3 subunit can undergo PKC-dependent phosphorylation after prostaglandin E2 receptor activation during peripheral inflammation (Harvey et al., 2004). This significantly reduces glycinergic drive in the DH and magnifies inflammatory pain signaling. Given this variability and impact of subunit composition at both GABAergic and glycinergic synapses, it would be useful for future work to assess the subunit composition of these inhibitory receptors on *Typical* and *Atypical* CR+ neurons with a view to selectively manipulating their activity.

The location of GABAergic and glycinergic DH neurons also differs in the DH. GABAergic neurons are more frequent in superficial laminae and glycinergic neurons dominate in deeper laminae (Todd and Sullivan, 1990; Todd et al., 1996, Polgár et al., 2013). This distribution is reflected in the nature of inhibitory IPSCs recorded in DH neurons and the impact blocking each transmitter system on laminar activation – GABAergic inputs dominates in superficial neurons while large glycinergic input is more prevalent in deeper neurons (Cronin et al., 2004, Anderson et al., 2009). Interestingly, although our study identified a difference in the relative contribution of glycine and GABA to inhibition of *Typical* and *Atypical CR+* neurons, both populations were located in the superficial DH (predominantly lamina II). Glycine dominates on putative inhibitory *Atypical CR+* neurons, whereas *Typical CR+* neurons received more mixed inhibition. Consistent with this finding we have also assessed the relative sources of inhibition to another inhibitory interneuron population in the DH that expresses the calcium binding protein, parvalbumin (unpublished observations). These neurons also receive inhibition that is dominated by glycine, but are concentrated more ventrally, in lamina III. Interestingly, the parvalbumin population also exhibited a tonic glycinergic current that plays an important role in regulating intrinsic excitability and action potential discharge. Other work has also described tonic glycinergic and GABAergic currents in inhibitory DH populations (Takazawa and MacDermott, 2010), however, we found no evidence of tonic currents in either the *Atypical* or *Typical* CR+ neurons. In the context of pathological pain, where one goal of therapies is to enhance spinal inhibition, our data suggest that enhancing glycinergic inhibition alone may be counterproductive as this would suppress activity in inhibitory (*Atypical*) CR+ neurons. Conversely, therapies that enhance GABAergic inhibition would preferentially affect the excitatory (*Typical*) CR+ populations and diminish spinal nociceptive signaling.

Our experiments on the postsynaptic responsiveness of the CR+ population to neuromodulators also provide insights into their function. Bath application of noradrenaline, serotonin, and enkephalin pronounced sustained outward currents in subsets of CR+ neurons. Interestingly, response profiles could be predicted by the classification of CR+ neurons as *Typical* or *Atypical. Typical* CR+ neurons responded to noradrenaline and serotonin but not enkephalin, and *Atypical CR+* neurons exhibiting the opposite response profile. This distinction reinforces the functional difference between *Typical* and *Atypical* CR+ neurons. A variety of studies have previously reported noradrenaline- and serotonin-induced outward currents in DH neurons and identified these currents as potassium channel-mediated (North and Yoshimura, 1984, Lu and Perl, 2007, Abe et al., 2009, Yasaka et al., 2010). Some of this work indicates that excitatory neurons are among those exhibiting outward currents in response to both monoamines, and are therefore inhibited. This work reported that a proportion of inhibitory neurons also exhibited monoamine-induced outward currents, implying that they too would be inhibited (Yasaka et al., 2010). This contrasts with our finding that *Atypical* (inhibitory) CR+ neurons are unaffected by noradrenaline and serotonin and adds further evidence that *Atypical* CR+ neurons are a distinct inhibitory subtype with a potentially unique role in spinal sensory processing circuits. Regarding the likely impact of these response profiles for spinal nociceptive circuits, both noradrenaline and serotonin have well-established roles in the descending antinociceptive regulation of pain (Millan, 2002). Our observation that these monoamines induced an outward current that would cause inhibition of Typical CR+ (excitatory) neurons, without affecting the Atypical CR+ (inhibitory) population, is compatible with this antinociceptive role.

In addition to descending monoaminergic control, we also assessed the postsynaptic responsiveness of CR+ populations to enkephalins, which contribute to the endogenous antinociceptive system (Millan, 2002). Previous work has shown that enkephalin, as well as more specific mu- and delta-opioid receptor agonists, are capable of inducing outward currents or associated membrane hyperpolarizations in approximately 40% of DH neurons (Hori et al., 1992, Schneider et al., 1998, Eckert et al., 2003). Furthermore, the ionic basis of these currents has been identified as opioid receptor activation of G-protein-gated inwardly rectifying potassium (GIRK) channels (Marker et al., 2005). Intriguingly, our experiments identified robust outward currents in *Atypical* CR+ neurons during bath application of enkephalin whereas *Typical* CR*+* neurons were unaffected. This finding, that we also replicated using mu- and delta-opioid receptor agonists, suggests *Atypical* CR+ neurons express both opioid receptor types. This response pattern agrees with similar work showing a selective postsynaptic responsiveness to enkephalin in DH neurons that exhibit tonic firing, an action potential discharge mode common to inhibitory interneurons (Yasaka et al., 2010) but not in putative excitatory neurons (Santos et al., 2004). Thus, enkephalin signaling in these populations would decrease signaling from *Atypical CR+* (inhibitory) neurons without altering *Typical CR+* (excitatory) neuron activity, a combination that is more difficult to reconcile given the analgesic action of the enkephalins. One factor that may help to explain differences in our enkephalin-induced neuronal responses in the context of the well-known behavioral actions of these ligands, lies in the postsynaptic target of *Atypical* CR+ neurons. For example, if *Atypical CR+* neurons regulate the activity of other inhibitory populations, reduced excitability during enkephalin activation would allow these downstream targets to exert their inhibitory actions in DH circuits. It is also important to recognize that enkephalins have well-described presynaptic effects in the DH, specifically depression of excitatory synaptic activity in primary afferents (Kohno et al., 1999, Ikoma et al., 2007). Thus, notwithstanding the predicted reduction in *Atypical* CR+ neuron excitability during enkephalin exposure, the widely acknowledged presynaptic effects of enkephalins may overwhelm the consequences of reduced *Atypical* CR+ neuron function to produce analgesia.

## Conclusions

The current experiments provide new insights into how CR+ neuron activity is regulated in spinal sensory processing circuits. It builds on our previous work describing two CR+ populations with distinct electrophysiological and anatomical properties. We now show that our *Typical*/*Atypical CR+* classification can predict the type of inhibitory drive and responsiveness to neuromodulators in each population. The different contribution of GABA and glycine to inhibitory inputs in the two populations, along with their differing responses to important neuromodulators, suggests that *Typical* and *Atypical* CR+ neurons have distinct roles in sensory processing. These findings have relevance for future work that seeks to selectively manipulate the activity of DH populations to alleviate chronic pain symptoms.

## Funding

This work was funded by the National Health and Medical Research Council (NHMRC) of Australia (grant 631000 and 1043933 to B.A.G), the BBSRC (grant BB/J000620/1 to D.I.H.), and the Hunter Medical Research Institute (grant to B.A.G. and R.J.C.).
